# CD44: A metastasis driver and therapeutic target

**DOI:** 10.18632/oncoscience.335

**Published:** 2016-12-30

**Authors:** Joseph L. Sottnik, Dan Theodorescu

**Affiliations:** Department of Surgery, University of Colorado, Denver, Aurora, CO 80045; Department of Pharmacology, University of Colorado, Denver, Aurora, CO 80045; University of Colorado Comprehensive Cancer Center, Aurora, CO 80045

**Keywords:** CD44, Osteopontin, metastasis, biomarker, therapy

CD44 is the canonical receptor for hyaluronic acid (HA) but is capable of binding other ligands such as osteopontin (OPN) [1-3]. CD44-HA interactions are important mediators of the inflammatory response associated with immune cell development, differentiation, and recruitment [4]. In addition to the standard CD44 receptor (CD44s), CD44 can undergo alternative splicing to form CD44 variants (CD44v) with an altered extracellular stem region which leads to additional interactions with surface bound receptor tyrosine kinases (RTKs) and signaling molecules on neighboring cells, leading to myriad physiologic processes [2].

**Figure 1:**
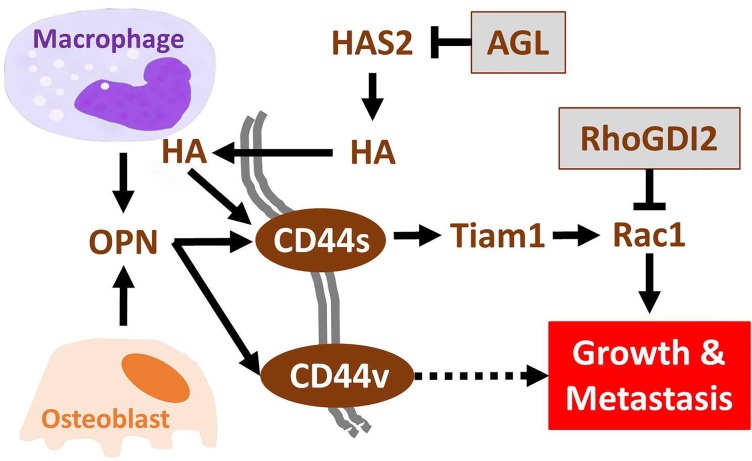
CD44 as a pleotropic receptor promoting tumor growth and metastasis

CD44 also plays a role in pathological processes such as cancer. We have shown the importance interactions between CD44 and hyaluronic acid (HA) in driving growth of tumors that have lost the Amylo-alpha- 1-6-glucosidase-4-alpha-glucanotransferase (AGL) gene which increases hyaluronic acid synthase 2 (HAS2) levels leading to higher HA levels (Figure 1) [3]. Similarly, the interaction between CD44 and OPN has been shown to promote bladder cancer growth and metastasis [1]. While in this paper we describe the pro-tumorigenic and pro-metastatic effects of macrophage secreted OPN [1] in cells with various levels of the metastasis suppressor gene RhoGDI2, it bears mentioning that OPN was originally described as being secreted from osteoblasts during bone formation. These observations, together with bone being a common site of metastatic development across a number of tumor types (e.g. prostate, bladder, and breast), suggest bone derived OPN may promote outgrowth of bone metastases from cells that express CD44.

Bone is continually remodeling to adapt to physical forces placed upon it, leading to a cycle of osteolytic and osteoblastic responses. OPN secretion is typically associated with osteoblastic responses. Even though osteoblastic responses are curtailed with age, resulting in diseases such as osteoporosis, OPN and other factors remain necessary for maintaining bone homeostasis. It is therefore reasonable to speculate that bone derived OPN may lead to metastatic colonization in bone by exploiting the OPN-CD44-TIAM1-Rac1 axis we describe (Figure 1) [1]. Osteoblast OPN expression is induced by inflammatory mediators such as high glucose, IL-1β, IFN-γ, and TNF-α associated with the tumor microenvironment [5]. Accumulation of macrophages from the bone marrow and circulation, which secrete OPN, would further enhance tumor growth in bone (Figure 1).

Our work describes OPN binding to CD44s on bladder cells whereas others have previously shown OPN binding to CD44v10 in leukemia's and lymphomas [6]. OPN-CD44v10 has been shown to promote leukocyte recruitment to inflammatory sites. Since CD44s and CD44v10 can both bind OPN, it would be of interest to understand if different biological phenotypes are observed due to activation of different CD44 isoforms. However, determining the responses to a single ligand associated with CD44s or specific CD44 isoforms has not been well described, likely due to the challenge of multiple CD44 splice variants being expressed concurrently.

New therapeutics are necessary for the treatment of metastatic bone disease, which is lethal in nearly all patients. Although a promising target, previous clinical trials with anti-CD44 antibodies failed typically due to unintended off target effects. For example, an anti-CD44v6 monoclonal antibody conjugated to Mertansine (Bivatusamab) was stopped due to severe skin reactions related to the unknown expression of CD44v6 in squamous cells [7]. Other failures have been the result of inhibition of leukocyte recruitment likely due to CD44v10 targeting as discussed above [6]. However, in our opinion, these failures do not eliminate the promise of CD44 as a therapeutic target but merely highlight that a more thorough understanding of CD44v expression is necessary both in tumor and normal cells. For example, CD44v's are present on ductal breast carcinoma cells but lacking on normal ductal breast tissue, suggesting a specificity that could be exploited therapeutically. Other examples include CD44v6 in bladder and head and neck squamous cell carcinomas, CD44v8-10 in gastric cancer, and CD44v9 in prostate cancer. Normal tissue typically expresses CD44s where as CD44v expression is higher in tumor tissue. Expression of multiple CD44v's in a single tumor may also be associated with an increased metastatic potential [2]. These data led to the genesis of novel anti-CD44v specific humanized antibodies that are now entering clinical trials.

RG7356 is an anti-CD44 humanized monoclonal antibody targeting the HA binding domain. RG7356 is thought to partially act by triggering an inflammatory immune response leading to macrophage recruitment and direct tumor cell killing by antibody-dependent cellular phagocytosis [4]. RG7356 appears to be well tolerated in a phase I dose escalation study in acute myelogenous leukemia (AML) and solid tumors [8]. In contrast, in our paper we described macrophage derived OPN as promoting tumor growth [1], yet these findings are not necessarily contradictory. We can speculate that in a pre-treatment setting, immunosuppressive M2 macrophages promote tumor growth through secretion of OPN. However, in the presence of a therapeutic such as RG7356, new M1 inflammatory macrophages may be recruited to the tumor leading to tumor growth inhibition. M1 macrophage recruitment may have the added benefit of limiting OPN secretion from M2 macrophages present in the tumor microenvironment, thus reducing pro-growth signals conferred by CD44 activation. Flipping the balance of macrophages from M1 to M2 is thought to be an important tenant of successful immunotherapy.

In conclusion, the expression of multiple CD44v by tumor cells, and the myriad ligands that bind CD44v, create significant complexity but also opportunity for therapeutic specificity. Current anti-CD44 therapeutics are exploiting specific expression of CD44v on tumor cells to decrease off-target toxicity. We believe CD44 remains an interesting and promising therapeutic target in cancer.
